# TALENs—an indispensable tool in the era of CRISPR: a mini review

**DOI:** 10.1186/s43141-021-00225-z

**Published:** 2021-08-21

**Authors:** Anuradha Bhardwaj, Vikrant Nain

**Affiliations:** Department of Biotechnology, Gautam Buddha University, Greater Noida, Uttar Pradesh 201312 India

**Keywords:** Genome editing, TALEN, CRISPR, ZFN, Meganuclease

## Abstract

**Background:**

Genome of an organism has always fascinated life scientists. With the discovery of restriction endonucleases, scientists were able to make targeted manipulations (knockouts) in any gene sequence of any organism, by the technique popularly known as genome engineering. Though there is a range of genome editing tools, but this era of genome editing is dominated by the CRISPR/Cas9 tool due to its ease of design and handling. But, when it comes to clinical applications, CRISPR is not usually preferred. In this review, we will elaborate on the structural and functional role of designer nucleases with emphasis on TALENs and CRISPR/Cas9 genome editing system. We will also present the unique features of TALENs and limitations of CRISPRs which makes TALENs a better genome editing tool than CRISPRs.

**Main body:**

Genome editing is a robust technology used to make target specific DNA modifications in the genome of any organism. With the discovery of robust programmable endonucleases-based designer gene manipulating tools such as meganucleases (MN), zinc-finger nucleases (ZFNs), transcription activator-like effector nucleases (TALENs), and clustered regularly interspaced short palindromic repeats associated protein (CRISPR/Cas9), the research in this field has experienced a tremendous acceleration giving rise to a modern era of genome editing with better precision and specificity. Though, CRISPR-Cas9 platform has successfully gained more attention in the scientific world, TALENs and ZFNs are unique in their own ways. Apart from high-specificity, TALENs are proven to target the mitochondrial DNA (mito-TALEN), where gRNA of CRISPR is difficult to import. This review talks about genome editing goals fulfilled by TALENs and drawbacks of CRISPRs.

**Conclusions:**

This review provides significant insights into the pros and cons of the two most popular genome editing tools TALENs and CRISPRs. This mini review suggests that, TALENs provides novel opportunities in the field of therapeutics being highly specific and sensitive toward DNA modifications. In this article, we will briefly explore the special features of TALENs that makes this tool indispensable in the field of synthetic biology. This mini review provides great perspective in providing true guidance to the researchers working in the field of trait improvement via genome editing.

## Background

### Genome editing

Genome editing is a procedure that allows for site-specific modifications to be made in the genome of any organism [1]. There are two main goals for genome manipulation: one is to learn how new genes function and what functions they play in cell regulation. The second major use of genome manipulation is the creation of alternative treatment options for a variety of genetic disorders [2]. The evolution of genome engineering using designer nucleases has brought a paradigm shift in the field of biotechnology. These tools have demonstrated promising results in various domains including medical and agricultural sciences [3]. Also, both TALENs and CRISPR are crucial in elimination of undesired genes [4]. The appealing agricultural applications of nuclease-based genome editing include improved varieties of crops with high yields and desired features like enhanced nutritional content, greater shelf life, better stress tolerance, disease, and pest resistance [5].

Genome editing is being applied in many plant and animal species. The use of ZFN in marine animals such as zebrafish and many live-stock animals has been shown, while TALEN and CRISPR systems are used in the cell lines of many cattle species such as cows, pigs, and chickens [6, 7]. Even genome editing has found its applications in many infectious and non-infectious diseases [8, 9]. In preliminary experiments, the knocking-in protocol was used to accomplish this aim. Gene editing techniques have been used to treat a host of genetic abnormalities in human cell lines and cancer models [3, 10, 11] These positive results suggest that gene editing strategies have huge potential to treat human genetic diseases like Duchenne muscular dystrophy, cystic fibrosis, sickle cell anemia, and Down syndrome [11]. CRISPR approaches have also demonstrated promise in the detection and treatment of fatal diseases such as AIDS and cancer [12]. However, off-target mutations have been identified as a significant risk of these developments, prompting the adoption of more rigorous standards and the completion of clinical research criteria prior to human germline editing [13].

**Fig. 1:**
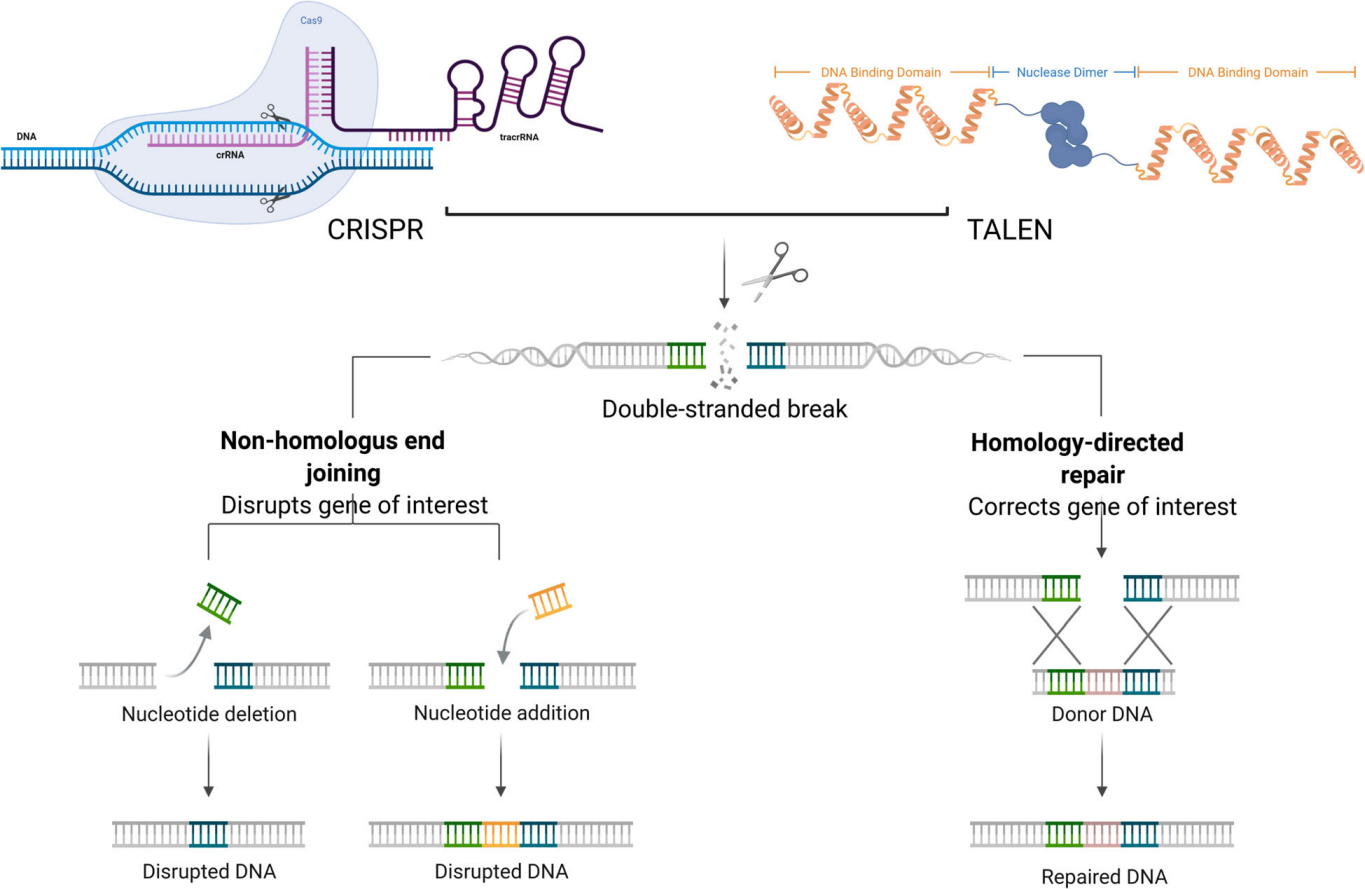
Schematic illustration of genome editing nucleases inducing double-strand break (DSB) in target DNA. DSBs activate the DNA repair pathways: the error-prone nonhomologous end joining (NHEJ) or accurate but template dependent homologous recombination (HR)

To modify a target gene, the genome-editing tools are designed to create a double-stranded break (DSB) precisely at the target specific region [14]. DSBs are particularly deleterious, but all living species have evolved repair mechanisms to restore the initial sequence in order to protect the functionality of their genomes. As soon as the DSB is created, either of the DNA repair pathways gets activated: (a) The error-prone non-homologous end joining (NHEJ) and (b) accurate homologous recombination (HR) (Fig. 1). NHEJ is preferred for gain/loss of function applications due to its mutagenic behavior of possible insertions or deletions (Indels) resulting in altered reading frames. In contrast, HR is cell cycle dependent (S/G2 phase) event having reduced efficiency of editing a genome due to its dependence on a template to accomplish repair. HR is usually preferred for gene knock-out/in experiments [15]. HR is usually preferred as its occurrence is governed by the cell cycle. Both of these mechanisms are extensively employed to manipulate genes.

**Fig. 2:**
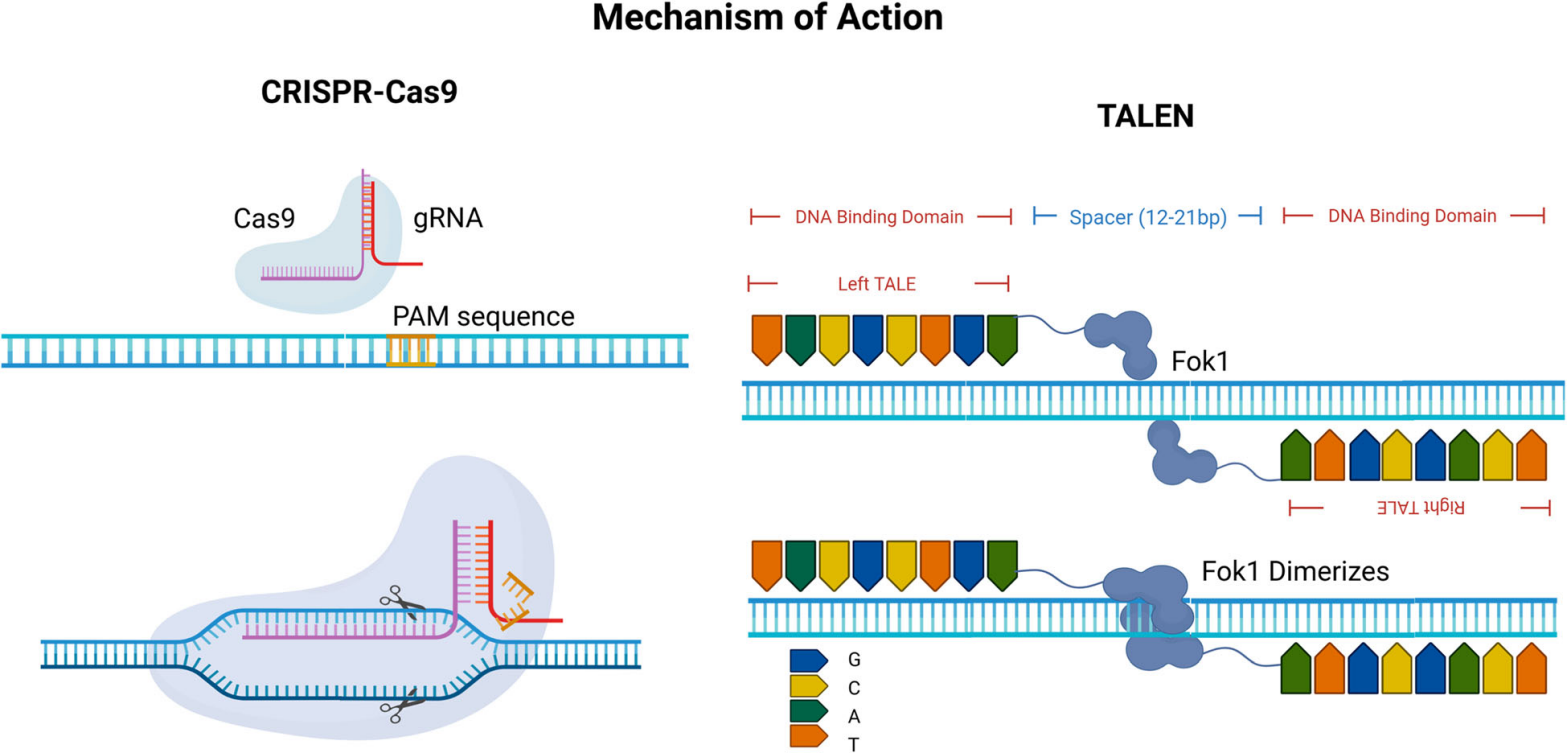
Schematic illustration of mode of action of genome editing nuclease (TALENs and CRISPRs) inducing a double-strand break on the target DNA

Out of three genome editing technologies (ZFN, TALEN, and CRISPR) available, TALENs and CRISPR are common in practice to make site-specific gene modifications [16, 17]. Figure 2 shows the mechanism of action of these genome editing tools TALENs and CRISPR/Cas9 system on a DNA double strand resulting in induction of DSBs and activation of different cell repair pathways. Both the tools manipulate genomes at the desired site, but they are structurally and functionally entirely different. While TALEN recognizes the target site on the basis of DNA protein interaction, CRISPR system is based on site specific RNA protein interactions [4]. This fundamental structural difference in TALEN and CRISPR leads to their strengths and weaknesses in terms of designing, synthesis, efficiency, specificity, and off target activity. In this mini review, we have discussed some of the unique features of TALENs that make it mighty in this epoch of CRISPR (Table 1).

**Table 1:** Comparison of TALEN and CRISPR/Cas9-mediated genome editing

Feature	TALEN	CRISPR/Cas9
Recognition type	DNA-Protein	DNA-RNA
Target site length	30-36 bp	23 bp
Endonuclease	Fok1	Cas9
Dimerization	Required	Not required
Off-target	Low	High
Design and Assembly	Labour intensive	Easy
Target Range	Unlimited	Limited by PAM
Degenerate Recognition	Yes	No
Specificity	High, few mismatches tolerated	Moderate, comparatively more mismatches tolerated
DNA methylation sensitive	Yes	No
Mitochondrial Genome Engineering	Easy	Complicated
Precision of Genome Editing	High	Moderate

## CRISPR

The CRISPR/Cas9 system for genome editing is considered the biggest scientific development of the decade, leading the Nobel prize to its inventors and opening up tremendous opportunities in the field of medicine and sustainable improvement in agriculture [18].The Cas9 (CRISPR-associated) enzyme, which is part of the type II CRISPR system that makes up *S. Pyogenes* bacteria’s innate immune system, has ushered this generation of genome engineering owing to its flexible use and easy construction. To configure Cas9 to knockout a given target DNA, the order of the guide RNA (gRNA) sequence must be designed to have a 5′ end complementary to the target site [19]. Cas9 targeting is simple to program compared to the more intensive genome editing tools (MNs, ZFNs, and TALENs). The endonuclease of the CRISPR-Cas9 system whose activity to specifically cleave DNA is governed by a short gRNA sequence (20bp). In Cas9 mediated targeting, the 5′-NGG-3′ PAM (protospacer-associated motif) interacting domain confers specificity and is therefore responsible for specifying the explicit binding site on target DNA. The Cas enzyme diversity is rich in various species that have different PAM requirements [20]. The CRISPR-Cas9 technology has been successfully operated in a variety of cells, organisms, and even human cells for medicinal purposes [21]. However, it is critical to note the high risk of off-target mutations as a significant shortcomings of the CRISPR/Cas9 platform [22].

### Limitations of CRISPR technology

Despite their wide range of applications, these designer nucleases are not thought to be safe or precise enough for site-directed therapies, particularly in gene therapy. Though off-target effects exist in all genome editing systems, the high prevalence (≥ 50%) of unpredictable off-targets in CRISPR/Cas9 technology is a major disadvantage [6]. Researchers have endeavored to mitigate CRISPR off-targets by creating novel Cas9 variants and improving the gRNA architecture [23], but these strategies have not been very efficient. Another disadvantage of this technology is the need for a PAM near the target location. With a short canonical PAM recognition site of 5′ NGG3′, where N can be any nucleotide, Cas9 from the bacteria Streptococcus pyogenes (SpCas9) is one of the most commonly used Cas9s. Furthermore, spCas9 is difficult to package into AAV vectors, the most popular gene therapy delivery vehicle, because of its bulky size [24]. CRISPR-induced DSBs often cause apoptosis resulting in DNA damage and cellular toxicity [25]. All traditional gene editing systems have technical flaws and functional drawbacks, raising concerns about their practical use in the real world.

## TALEN

TALEs proteins were first reported in 2009, derived from phytopathogenic bacterial genus *Xanthomonas* [26]. TALE is a special class of proteins that can bind DNA. TALEs offers flexible applications in genetic engineering due to its compatibility with many functional domains. Different associations of TALE proteins with transcriptional activators, repressors, or endonucleases give them potential transformation from transcriptional modulators to genome editing tools [27]. In the year 2011, the TALEN system was voted as the tool of the year by Nature Methods [28] due to its high specificity and precision in genome engineering making it an efficient tool.

A typical TALEN unit comprises a central DNA-binding domain of 12-28 repeats, a nuclear localization signal (NLS), an acidic domain for target gene transcription activation, and Fok1 nuclease [28]. The DNA-interacting region is a preserved sequential arrangement of significantly constant 33-35 amino acids with polymorphic 12 and 13 repeat variable di-residues (RVDs). Each repeat uniquely binds to a single nucleotide in the 5′ to 3′ orientation on the target [29]. The biochemical structure-function studies suggest that the amino acid present at the place 13 uniquely identifies a nucleotide on the DNA target major groove [30–32]. This DNA-protein interaction unit is stabilized by the amino acid at place 12. At the 3′-end of target locus, a half repeat of only 20 amino acids exists to bind the DNA sequence.

The four most common RVDs identified by various experimental validations are NN, NG, HD, and NI with unique preferential binding affinity toward G/A, T, C, and A respectively bestowing target specificity [33]. Remarkably screening of all 400 possible combinations of RVDs is also reported [34], which are considered as non-conventional RVDs because of their rare existence in nature [35].

The popularly used TALEN system comprises 2 units of DNA binding domain (DBDs) from TALE proteins. Each unit is attached with a catalytic domain from Folk1 restriction enzyme. Fok1 nuclease of the TALENs dimerizes which generates a cleavage on both the strands of DNA-double helix, activating the DNA repair machinery to fix disruption.

As we align repeat modules (RVDs) in a particular structure, it is possible to create TALENS with the required sequence precision. There is, however, a limit to the option of target sites for TALEN. A thymine at position 0, i.e., immediately precursory to the TALE-repeat bound sequences is invariably required [32]. The complete gene activation is ensured by the weak van der Waal forces acting between the C5 methyl group of thymine and the extremely conserved tryptophan in the N-terminal. Newer versions of TALEs are also reported in nature that replaces thymine with cytosine at position 0 with no effect on their activity. These scaffolds are independent of the prerequisite 5′T [36]. Nonetheless, customizing TALE-based tools to alter any genome is quite versatile but simple. The crystal structure of TALE proteins bound to target DNA reveals that each repeating unit forms a v-shaped structure consisting of two alpha helix assembled to form a solenoid-like structure wrapped around the major groove of DNA via the hypervariable 12 and 13 amino acids [31, 32].

### Characteristic features of TALENs

#### Specificity

This era of gene targeting is ruled by the two very recent and robust engineering nucleases, TALEN and CRISPR. CRISPR-Cas9 is a very familiar tool for many molecular biologist greatly known for its easy-programming of gRNA feature [37]. Since nucleases can cause unintended interruptions in the genome, gene editing is crucial and as multiplex methods become more widely used, the likelihood of off-targets and the downstream consequences of such off-target activity grow. Minimizing these undesired cleavage (off-targets) is a matter of utmost importance for any genome–engineering applications, especially in the therapeutic domain. Unwanted double-stranded breaks in the genome may lead to chromosome translocation, and cellular toxicity [38]. There are currently a wide variety of techniques available to predict and resolve off-target behaviors by analyzing secondary target locations [39–43].

The gRNA of CRISPR is an integrated product of custom-designed crRNA and trRNA scaffold [19]. On the target DNA, the sgRNA specifically guides the Cas9 of *S. pyogenes* to recognize its unique Protospacer Adjacent Motif, or PAM immediately adjacent to a 20-nucleotide target site where gRNA hybridizes (Watson-crick base pairing) with the strand opposite the PAM site channelizing Cas9 to cut DNA. CRISPR has been shown in a number of recent papers to induce DSB formation at very high frequencies at the desired DNA locus [44].

On the other hand, TALEs have a well-defined DNA base-pair choice, offering a basic strategy for scientific researchers and engineers to design and construct TALEs for genome alteration. Engineered TALES can currently be used in cells and model organisms. A protein repeat tandem is responsible for recognizing individual DNA base pairs. Tandem repeats are made up of a pair of alpha helices linked by a loop of three-residue of RVDs in the shape of a solenoid. For the creation of TALEs with variable precision and binding affinity, the six conventional RVDs (NG, HD, NI, NK, NH, and NN) are frequently used. HD and NG are associated with cytosine (C) and thymine (T) respectively. These associations are strong and exclusive [45].

NN is a degenerate RVD showing binding affinity for both guanine (G) and adenine (A), but its specificity for guanine is reported stronger. RVD NI binds with A and NK binds with G. These associations are exclusive but the binding affinity between these pairs is less due to which they are considered weak. Therefore, it is recommended to use RVD NH which binds with G with medium affinity. It is also worth noting that the binding affinity of TALE is influenced by the methylation status of the target DNA sequence [45].

A typical TALEN system usually consists of 18 repeats of 34 amino acids. A TALEN pair must bind to the target site on opposite sides, separated by a “spacer” of 14-20 nucleotides as an offset since FokI requires dimerization for operation. As a whole, such a long (approximately 36 bp) DNA binding site is predicted to appear in genomes very rare.

Remarkably, the high degree of specificity and low cytotoxicity of TALENs has been applied in diverse cell types [46]. Despite having more efficiency than TALEN, CRISPR-Cas9 system is prone to off-targets effects [47–51]. Most importantly, off-targets are a rare event in TALEN-mediated systems which make it a better choice for the purpose of genome editing [52]. This high level of specificity becomes important when we have to target a particular gene belonging to a gene family or a specific allele in a polyploid plant species.

#### Degeneracy

The TALEN code is degenerate, which means that certain RVDs can bind to multiple nucleotides with a diverse spectrum of efficiency. The binding ability of the NN (for A and G) and NS (A, C, and G) repeat variable di-residue empowers the TALENs to encode degeneracy for the target DNA [26]. This degeneracy may be useful in targeting hyper variable sites. TALENs technology is the only known genome editing tool which can be engineered in a way that can be easily used for the escape mutations in a genome [26]. This unique feature of TALENs make them a more flexible and reliable tool in the field of genome editing specifically in clinical applications to tolerate predicted mutations [53].

#### Methyl sensitivity

Apart from the four typical nucleotides A, T, G and C, the epigenetic DNA nucleobases 5-methylcytosine(5mc) and 5-hydroxymethylcytosine(5mhc) plays important regulatory roles, contributing to around 75% of CpG islands in a mammalian genome [46, 54]. They are key molecules in diverse biological processes ranging from development-related events like gene expression and gene silencing to a diseased state like X chromosome inactivation. CRISPR-Cas9 is binding place base-pairing between gRNA and the target DNA, which makes it insensitive to distinguish the methylation modifications of cytosine. A recently demonstrated method using the catalytically dead Cas9 (dCas9) fused to the DNA methyltransferase domain for detection of the methylation of CpG dinucleotides has been reported [55–57]. This method has lower efficiency and results in multiple CpGs methylation in the vicinity of the target site. In the case of a CpG island, low sequence complexity and targeting ambiguities can complicate guide design. A drawback of this method is that it fails to distinguish between the effects of the binding of the fusion protein vs. methylation itself for investigations of the functional consequences of methylation.

On the other hand, TALE proteins can be successfully re-engineered with sensitivity toward these DNA chemical modifications. Methylated cytosine is not efficiently bound by the canonical RVD HD; however, owing to the high structural resemblance of methylated cytosine to thymine, NG is capable of binding to methylated cytosine [58]. Also, for a given target DNA, engineered TALEs with a combination of HD for C, N* for 5mC, Q* for 5mhC, are used as DNA binding receptors to directly distinguish methylated cytosines (5mC, 5mhC) from unmethylated cytosine (C) [59–61] Even in the case of methylated cytosines, the peculiar characteristic of degeneracy in TALENs exists. RVD R*, for example, codes for both 5mC and 5mhC epigenetic nucleobases, while RVD Q* is a universal RVD that associates with both C, 5mC, and 5mhC epigenetic nucleobases making it a powerful and versatile tool for genome editing [62].

#### Cell organelle DNA targeting

CRISPR-Cas9 is known for its broad spectrum of successful applications in this modern era of nuclear DNA editing, but researchers find it challenging to import the gRNA and Cas9 complex to reach into the mitochondria to selectively eliminate mutations. The CRISPR/Cas9 platform, tailored to mitochondria, could prompt a revolution in mitochondrial genome engineering and biological understanding [63]. However, the presence of an endogenous system, a prerequisite for mitochondrial CRISPR/Cas9 gene editing, for nucleic acid induction into mammalian mitochondria, remains unclear [64]. TALEN, on the other hand, has the ability to efficiently manipulate mtDNA (mitochondrial DNA) as a treatment for treating human mitochondrial diseases triggered by mitochondrial pathogenic mutations [54]. Mito-TALENs (mitochondrial-targeted TALENs) have also been proven to be effectively treating human mitochondrial disorders affected by mtDNA mutations, such as Leber’s hereditary optic neuropathy, ataxia, neurogenic muscle fatigue, and retinal pigmentosa [55]. Plastid engineering has also demonstrated competent results in limited varieties of plants for crop improvements [65].

#### Development of artificial transcription factors

Control over the endogenous gene expression has always fascinated scientists. Programmable designer transcription factors fused to desired transcriptional activator and repressor protein domains provides this flexibility to the researchers to keep a strong hold on transcriptional machinery of a gene. Before their application in genome modifications, proteins like ZFN and TALE have demonstrated a wide range of applications in this sector of modulating the expression of any gene of any organism [66, 67].

Most recently, a novel modified version of the easy to design non-functional Cas9 based CRISPR-Cas9 system was also developed to be used as an artificial transcription factor; however, due to the large size of the complex, it is not as efficient as TALE protein-based artificial transcription factors.

#### Development of transcription activators

Artificial transcription factors were first generated by the fusion of engineered zinc finger protein with a 16 amino acid peptide (VP16) from herpes simplex virus as a transactivation domain [68]. With a glycine-serine linkage between four consecutive VP16, a novel assembly of synthetic transcription factor VP64 was then assembled [69]. However, this tool suffered a problem of high incidence of off-targets. As a modified version of zinc-finger transcription factors, TALE transcription factors have emerged as a crucial tool for achieving selective transcriptional regulation [70]. In contrast with zinc-fingers, TALEs are efficient transcription modulators with only 10.5 repeats with an effector module fused to the carboxyl terminal [71]. TALEs in the form of activators have been used to control the gene expression in case of external stimuli like a chemical change, or optical stimulus [72]. TALE transcription factors are used in various organisms including plants and animals.

To explore CRISPR/Cas9 as a transcription modulator, the Cas9 protein of the system is catalytically deactivated and then fused with the desired effector domains like VP64 [73]. The inactivation of Cas9 protein to form dead/deactivated Cas9 (dCas9) is accomplished by D10A and H840A amino acid substitutions in the RuvC and NHN endonuclease domains, respectively. Though catalytically inactive, dCas9 retains its specificity to bind at the gRNA directed site on the target [55, 74].

#### Development of transcription repressors

Earlier in its old versions, CRISPR/Cas9 acts as a repressor modulator of transcription by blocking the target site. The steric hindrance of the CRISPR system hindered the transcription machinery to affect the target on the DNA. In the modified version, the involvement of the repression domain was more efficient than only steric repulsions. In the revised versions, a mechanism known as CRISPR interference in which the dead Cas9 protein blocks the gene expression by obstructing the transcription start site is also in practice. Fusing dCas9 to transcriptional repressor domains is another way to effectively silence a gene from the promoter [75]. To accomplish repression of a gene, the robust TALE proteins are attached with either Kruppel-associated box (KRAB), Sid4, or EAR-repression domain (SRDX) repressors [76].

#### Editing the epigenome

Natural modifications in the cytosine base are absolutely necessary to maintain regulation of genome expression and genome stability. ATFs are programmed to engineer the epigenome to modulate the expression of a gene without altering the DNA sequence. These epigenome editing techniques help us explore the role of epigenetics in the crucial process of gene expression. Moreover, epigenome editing uncovers the exact sequence of events of chromatin remodeling and its effect of gene expression, which is key to understanding many biological processes and diseases in humans. In comparison with ZFNs, TALE-proteins have significantly emerged as critical DNA-binding scaffolds governed by a simple cipher. Their compatibility with a broad range of epigenetic modifiers is commendable [77]. With these DNA-binding proteins, it is possible to target an epigenetic effector domain to any locus in the genome [78].

#### Modifying histones

To understand the function of histone modifications, zinc-finger proteins were fused with a methyltransferase in a research by Carl who successfully demonstrated that the methylation of H3K9 can ultimately result in gene repression [79]. The lysine-specific histone demethylase 1 (LSD1) domain is sensitive in detecting histone demethylation. A hybrid TALE with LSD1 has been successfully employed to bring out histone modifications to reveal its function [80]. The lab of Feng Zhang has created an optogenetic epiTALE system in which an inducible element both activates and represses transcription via effector domains, many of which modify histones [81]. CRISPR/dCas9 fused with a p300 effector domain creates to activate enhancers by histone acetyltransferase.

#### DNA—cytosine demethylases

A wide range of cytosine modifications naturally exists like 5methylcytosine (5mC) and 5 hydroxymethyl (5hmC). Unwanted DNA methylations are associated with many neurodegenerative diseases. The catalytic domain thymidine DNA glycosylase (TDG) was first demonstrated to abolish the DNA methylation and induce gene expression [82]. In the clinical practices, the hybrid zinc finger protein with TET domain (ten-eleven translocation methylcytosine dioxygenase 2) has demonstrated a great potential for targeting epigenetically silenced cancer gene (ICAM-1) and induce its expression in cancerous cells [73, 82]. The CRISPR-dCas9 fusion with TET1 has been successfully used in the treatment of many diseases like diabetes (inducing β cell replication) and cancer (inhibiting cell proliferation) [83, 84].

#### Delivery of TALENS vs CRISPR

For the real-world clinical applications of the genome editing systems, it is important to develop efficient in vivo delivery strategies to improve safety. The traditional in vivo delivery methods are either viral vector based or non-viral vector based. Viral vectors such as adeno-associated virus (AAV), adenovirus (AdV), and lentivirus (LV), are well established to carry and deliver small ZFNs and even TALENs into target cells, but are not generally preferred for the CRISPR-Cas9 system due to its bulky architecture and large cargo size (~4.3kb) (effect of genome size on AAV vector packaging). Viral vectors are popular for the delivery of nucleases in the cells with well-established protocols as they do not cause insertional mutagenesis and have higher efficiency. Though, it is reported that the LV-encapsulated delivery vector of CRISPR-Cas9 system for gene therapy produced efficient-targeted mutations but higher risk of off-targets limits its applications. In contrast, the easily designed and constructed non-viral vectors have capability to carry large size designer nucleases but their low transfection efficiency and poor specificity causes high cellular toxicity which limits their use in gene therapy [85].

## Conclusions

TALEN is a robust and promising genome editing tool which offers the scientific community a wide choice to target unlimited sequences of any organism. It is a powerful tool with high specificity and precision with low cytotoxicity which makes it ideal for therapeutic applications especially in humans. It is easy and relatively cheap to design and assemble TALENs and thus it becomes the first choice for targeted mutagenic applications. The potentials of TALENs are neglected over CRISPR due to its ease of design but, when it comes to the real world problems, the outstanding competence of TALENs is undoubted.

## Data Availability

Not applicable

## Abbreviations

MN: Meganucleases
ZFNs: Zinc-finger nucleases
TALENs: Transcription activator-like effector nucleases
CRISPR/Cas9: Clustered regularly interspaced short palindromic repeats associated protein
DSB: Double-stranded break
NHEJ: Nonhomologous end joining
HR: Homologous recombination
gRNA: Guide RNA
